# Applying the theory of planned behaviour to explain HIV testing in antenatal settings in Addis Ababa - a cohort study

**DOI:** 10.1186/1472-6963-11-196

**Published:** 2011-08-18

**Authors:** Alemnesh H Mirkuzie, Mitike M Sisay, Karen Marie Moland, Anne N Åstrøm

**Affiliations:** 1 Centre for International Health, Faculty of Medicine and Dentistry, University of Bergen, Overlege Danielsens Hus, Årstav. 21, Postbox 7804, Bergen 5020, Norway; 2 College of Medical and Health Sciences, Department of Nursing and Midwifry, Hawassa University, Awassa, p.o.box 1560, Ethiopia; 3 Behavioural Sciences Unit, School of Public Health, Collage of Health Sciences, Addis Ababa University, Black Lion Hospital, P.O.Box 9086 Addis Ababa, Ethiopia; 4 Department of Nursing, Bergen University College, Haukelandsbakken 45, Bergen 5009, Norway; 5 Department of Clinical Dentistry, Faculty of Medicine and Dentistry, University of Bergen, Årstav. 17, Bergen 5020, Norway

## Abstract

**Background:**

To facilitate access to the prevention of mother-to-child HIV transmission (PMTCT) services, HIV counselling and testing are offered routinely in antenatal care settings. Focusing a cohort of pregnant women attending public and private antenatal care facilities, this study applied an extended version of the Theory of Planned Behaviour (TPB) to explain intended- and actual HIV testing.

**Methods:**

A sequential exploratory mixed methods study was conducted in Addis Ababa in 2009. The study involved first time antenatal attendees from public- and private health care facilities. Three Focus Group Discussions were conducted to inform the TPB questionnaire. A total of 3033 women completed the baseline TPB interviews, including attitudes, subjective norms, perceived behavioural control and intention with respect to HIV testing, whereas 2928 completed actual HIV testing at follow up. Data were analysed using descriptive statistics, Chi-square tests, Fisher's Exact tests, Internal consistency reliability, Pearson's correlation, Linear regression, Logistic regression and using Epidemiological indices. P-values < 0.05 was considered significant and 95% Confidence Interval (CI) was used for the odds ratio.

**Results:**

The TPB explained 9.2% and 16.4% of the variance in intention among public- and private health facility attendees. Intention and perceived barriers explained 2.4% and external variables explained 7% of the total variance in HIV testing. Positive and negative predictive values of intention were 96% and 6% respectively. Across both groups, subjective norm explained a substantial amount of variance in intention, followed by attitudes. Women intended to test for HIV if they perceived social support and anticipated positive consequences following test performance. Type of counselling did not modify the link between intended and actual HIV testing.

**Conclusion:**

The TPB explained substantial amount of variance in intention to test but was less sufficient in explaining actual HIV testing. This low explanatory power of TPB was mainly due to the large proportion of low intenders that ended up being tested contrary to their intention before entering the antenatal clinic. PMTCT programs should strengthen women's intention through social approval and information that testing will provide positive consequences for them. However, women's rights to opt-out should be emphasized in any attempt to improve the PMTCT programs.

## Background

Behavioural change interventions such as sexual risk reduction and persistent condom use remain critical HIV prevention strategies, when finding a cure or vaccine against the virus is challenging [1,2]. Being part of the HIV/AIDS prevention strategy, the prevention of mother- to-child HIV transmission (PMTCT) programme aims to curb vertical transmission of HIV from mothers to their infants. Arrays of interventions that are recommended for PMTCT include HIV counselling and testing, antiretroviral (ARV) prophylaxis, safe obstetric practices and safe infant feeding counselling [3]. The HIV counselling and testing intends to screen HIV positive pregnant women for subsequent PMTCT interventions. Until 2004 HIV testing was offered solely in an opt-in approach in resource poor settings. In this approach the testing is initiated by the client and assumed to be voluntary. However, due to some social and structural barriers, acceptability and rate of HIV testing continued to be suboptimal [4,5]. This prompted the WHO to call for a shift in HIV testing approach [6]. Subsequently, routine opt-out HIV testing has become the standard of care for all pregnant women.

Following the implementation of routine opt-out approach a significant improvement in acceptability has been reported with rates of testing ranging from 55% to 100% [4,7-9]. There is however some evidence that the pre-test counselling and the right to opt-out are being compromised. Studies from Kenya and Uganda showed that the information about the possibility to opt-out was not well communicated during the group pre-test counselling [10,11]. Many researchers also argue that most pregnant women in resource poor settings are less empowered and are thus less likely to resist the pressure to comply with the advice given by health professionals in antenatal settings [11-13]. Some women may also get tested against their intention not to do so, believing that testing is a necessary condition to access subsequent care [12,14]. Several researches concerning routine opt-out testing are primarily focused on the tension between increasing the rate of testing and the potential violation of ethical principles [10,11,13,15-17]. Yet, few studies have considered this issue using cognitive-behavioural approach.

**Figure 1:**
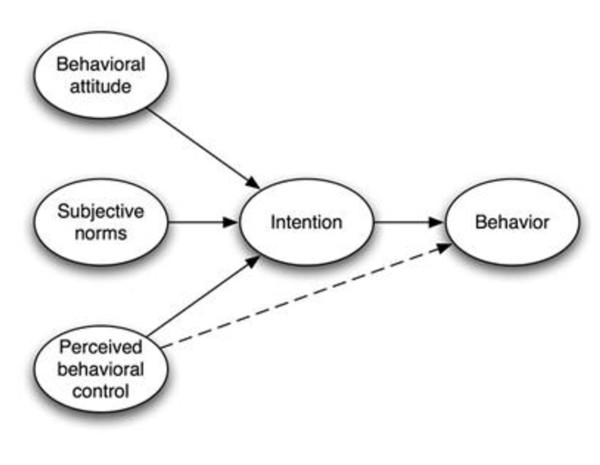
**Theory of planned behaviour**.

According to Jessor (1997), the factors influencing any behaviour, including HIV related behaviour might be ordered along a dimension of conceptual proximity to immediate experience with the particular behaviour [18]. Distal influencing factors including cultural and socio-demographics are largely operating through or are mediated by cognitive processes (proximal determinant) [19]. Taking this point of departure, the more one knows about the cognitive influencing factors of a particular behaviour, the easier it will be to change that behaviour. Being a theory of the proximal cognitive determinants of behaviour, the TPB constitutes a promising theoretical framework for explaining and predicting social behaviours [1] (Figure 1). The theory also seems to constitute a practical tool for the analysis of HIV testing behaviour in antenatal settings and for the identification of barriers and facilitators to change of behaviour.

The TPB includes perceived behavioural control (PBC) on a level with attitude and subjective norm as predictors of behavioural intention, implying that the three predictors influence subsequent behaviour indirectly through behavioural intention (Figure 1). In turn, intention is the key proximal predictor of behaviour. According to the TPB, behavioural intention is a function of attitude, reflecting a favourable or unfavourable evaluation of the particular behaviour and subjective norm. The subjective norm here is referring the perceived social pressure to perform the behaviour. Perceived behavioural control reflects the ease or difficulty associated with performance. Attitudes, subjective norms and perceived behavioural control are underpinned by behavioural, normative and control beliefs, respectively.

A considerable body of research has confirmed the power of the TPB to predict intentions and behaviours across a range of health behaviours [20]. In a meta-analysis, Armitage and Conner (2001) reported that attitude, subjective norms and PBC accounted for 39% of the variance in intention across 154 applications, whereas intention and PBC accounted for 27% of the variance in behaviours across 63 applications [21]. Nevertheless, some studies have reported the explanatory power of the TPB to be as low as 13% [21] and 7% [22]. Previously, the TPB has been used in sub Saharan African settings to predict HIV preventive behaviours [23-27], mostly in small scaled studies of cross-sectional design. However, the strength of the intention-behaviour relationship in the domain of routine HIV testing using objectively assessed behaviours in prospective studies still remains to be demonstrated.

Empirical works have shown that variables outside the TPB can capture a substantial proportions of variance in explaining intention and behaviour [1,28]. Of these variables past behaviour and descriptive norms had residual effects on intention and behaviour after the TPB variables have been taken into account [26-29]. Descriptive norm here is referring to women's perceptions of what other antenatal attendees (friends, sisters, neighbours...) do with respect to HIV testing. Focusing a cohort of pregnant women attending public and private antenatal care facilities for the first time in their current pregnancy, this study set out to explain intended and actual HIV testing using the extended version of the TPB framework. According to the TPB, attitudes, subjective norms and perceived behavioural control would predict intended HIV testing, whereas intention and perceived behavioural control would predict actual HIV testing. It was proposed further that the external variables in terms of socio-demographics, descriptive norm, PMTCT knowledge and previous HIV testing experience would add to the explanation of intended- and actual HIV testing beyond the TPB variables. Finally, this study examined whether type of pre-test counselling would modify the strength of the intention behaviour relationship.

## Methods

### Study settings

The present study was conducted in Addis Ababa, the capital of Ethiopia. The city is administratively divided into 10 sub-cities. The HIV prevalence among adults (15-24 years) in the city is estimated to be 8.8% were the majority of the infections occur through hetro-sexual contact [30]. In total 54 health facilities were providing PMTCT services, where 25 were public health centres. The health services were fairly accessible with a median distance to the nearest referral centre being less than 5 km [31]. About 90% of the pregnant women in the city had antenatal visit at least once and about 90% of these attended public health facilities [30,32]. In 2009 alone 54 698 women attended PMTCT programmes across the city, about 79% received HIV counselling and testing and 4.6% were HIV positive [33].

The first national PMTCT guidelines were developed in 2001 and incorporated an opt-in HIV counselling and testing approach [34]. Two years following the development of the guidelines, PMTCT programmes were launched in selected public health facilities across the country. In 2007, when the PMTCT guidelines were revised, the HIV testing approach shifted from opt-in to routine opt-out [35]. From early 2008, the routine opt-out HIV testing has become the standard of practice in public health facilities [9]. In these facilities, pregnant women were offered HIV testing routinely following an individual or group pre-test counselling free of charge. The PMTCT counsellors attending these women did the HIV testing in the antenatal clinics and the test result often made available in 30 minutes. In private health facilities by contrast, opt-in HIV testing remained the standard of practice since the launching of the PMTCT programmes in 2007. In these facilities, pregnant women received antenatal care by a medical doctor, and were then referred to another room for HIV testing. Pregnant women were paying service charges when they attended private health facilities.

### Study design and participants

A sequential exploratory mixed methods study was conducted in January-February, 2009. In this study the qualitative Focus Group Discussions (FGDs) were followed by a prospective cohort study. In a mixed methods design, one of the study designs could have a primary role while the other design has a supportive role [36]. In this study, the cohort was the main design supported by the FGDs. Data were collected in three phases. In the first phase, three FGDs were conducted by the principal investigator in three different health care facilities among first time antenatal attendees. Twenty seven women were selected purposefully for the FGDs. Prior to each FGD all women were explained about the study aim and consents were inquired. During the discussions women were first asked about their demographic and obstetric information then the discussion about HIV testing followed. The questions were "what factors or circumstances would enable you/make it difficult for you to test for HIV upon first time antenatal attendance?", and " are there any other issues that come to mind when you think of HIV testing upon first time antenatal attendance?". The participants identified three potential barriers to HIV testing including "scared to test", "concerns about confidentiality" and "fear of disclosing HIV positive results". The participants were also probed as to whether "fear of stigma and/or discrimination", "fear of being chased from home in the case of positive test result" and "fear that they would be denied of proper care" would affect their decision to test. Of the probed questions only "fear of discrimination" was mentioned as a potential barrier to HIV testing. All the potential barriers identified by the participants were included in the questionnaire and used to measure the concept of perceived behavioural control. None of the FGD participants were included in the subsequent questionnaire interviews.

In the second and the third phase, data were collected from women attending antenatal care in 12 public health centres and three private hospitals. A four-to-one public to private ratio was used in selecting health facilities, considering the fact that over 80% of the pregnant women in the city received care from public health facilities. Then individual health facilities were selected on the bases of high client flow and to have representation of all the 10 sub-cities. The inclusion criteria were attending antenatal care for the first time in the current pregnancy, attending selected health facilities, attending during the study period and consenting to be followed up. Known HIV positive women were excluded as they were not eligible for further testing. The study covered 44.3% of the eligible women attending antenatal care across the city. The cohort used a fixed follow up period.

In the second phase of data collection, a survey was conducted using a pre-tested structured TPB questionnaire to measure the concepts of attitudes, subjective norms, descriptive norms and behavioural intention. The questionnaire was first translated into Amharic (the official language fluently spoken by all participants) then back translated to English for validation. Two days training was given to 17 field assistants (college students) who participated in pre-testing of the questionnaire under close supervision of the principal investigator. The questionnaire was administered in a face-to-face interview in the maternity waiting area before the women received pre-test counselling. The interviews were conducted in private while the women waited for their turn for antenatal check up one after the other in a quite corner of the waiting areas. For ethical reasons we used the women's antenatal number as a unique identifier in the questionnaire. Completed questionnaires were then handed to PMTCT counsellors providing antenatal care and/or PMTCT services at the end of each day. Among the 3082 women approached, 1.5% (49) refused to participate while 3033 had completed the questionnaire interviews. Intention to test for HIV was the intermediate outcome in the second phase.

**Figure 2:**
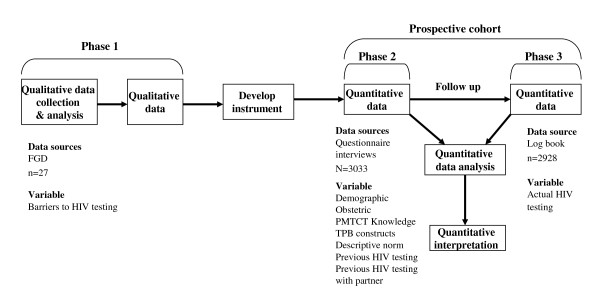
**The sequential mixed methods design, the three phases of the study and the study participants in each phase**.

In the third phase, the follow up data were collected by the PMTCT counsellors from PMTCT log books. The PMTCT counsellors were routinely conducting the HIV testing and registering whether the woman received pre-test counselling (group or individual), testing, post-test counselling and the HIV test result in the log books. These questions were also included in the questionnaire. The registration was done anonymously using the women antenatal number as a unique identifier. To be consistent, these unique identifiers were used in the questionnaires. Thirty three PMTCT counsellors were trained for two hours on how to collaborate with the field assistants and on how to match each questionnaire with the information obtained from the PMTCT logbooks using the unique identifiers and to report inconsistencies. Two hours training were found adequate as they were the ones who did register HIV counselling and testing information routinely in logbooks. Furthermore, the principal investigator supervised them when they obtained follow up information for the first time. Inconsistencies were validated by crosschecking with the woman's antenatal folder. The study team were checking and collecting the questionnaires every day. Of the 3033 women who completed the TPB interview in the second phase, 2928 were assessed objectively whether they actually tested for HIV or not during the follow up (Figure 2). The final outcome measured in this third phase was actual HIV testing.

The study was reviewed and approved by the Ethical committee of Addis Ababa City Administration Health Bureau in Ethiopia and the Regional Committee for Medical Research Ethics in Western Norway. Study permits from Addis Ababa City Administration Health Bureau and respective sub cities were obtained. Informed verbal consent was inquired before each interview.

### Study variables

The second phase structured interview covered demographic- and obstetric information, PMTCT knowledge, TPB variables and questions related to previous HIV testing. The TPB variables were assessed in relation to testing for HIV upon first time antenatal care attendance. A five point response scale was used (1) 'very likely' to (5) 'very unlikely', (1) 'very certain' to (5) 'very uncertain' and from (1) 'strongly agree' to (5) 'strongly disagree'. A sum score was constructed by adding the items corresponding to each variable. The higher the score the more positive the attitude, the stronger the intention, the stronger the subjective norm and the more barriers perceived with respect to HIV testing. Intention to test for HIV was assessed using three items; 'I intend/I plan/I want to test for HIV upon first antenatal attendance'. A sum score was constructed by adding the three items. For use in logistic regression model later, intention was dichotomized. Women who scored greater than or equal to the mean score were considered to have high intention; otherwise they were regarded to have low intention. Attitude towards HIV testing was assessed using three items; 'For me, testing for HIV upon my first antenatal attendance is beneficial/right thing to do/bad'. A sum score was constructed by adding the three items. Subjective norm was assessed using four items; 'People who are important to me think that I should test for HIV upon my first antenatal attendance, 'People who are important to me would appreciate that I tested for HIV...', 'My husband agreed that I should test for HIV ...' and 'People who are important to me encouraged me to test for HIV ...'. A sum score was constructed by adding the four items. Descriptive norm was assessed using one item. 'Women who I know and who are important to me would themselves test for HIV upon their first antenatal attendance'. Perceived barrier was assessed using four items; 'For me to test for HIV upon first antenatal attendance is difficult because I feel scared to know the result/I do not want to disclose my HIV status/I suspected that test result will not be kept confidential/People may discriminate me if I found HIV positive'. A sum score was constructed by adding the four items. For use in a logistic regression model later, perceived barrier was dichotomized. Women who scored greater than or equal to the mean score were considered to have perceived barriers to test for HIV; otherwise they were considered to have no perceived barriers.

Previous HIV testing experience was assessed using two questions, i.e 'How many times have you been tested for HIV?' and 'How many times have you been tested for HIV with your partner' with a response range from 0 to more than 3 times. The correlation between the two measures was less than 0.7. Then we decided not to combine them into a single score but retain them as separate measures. For use in logistic regression model later, previous HIV testing experience was dichotomized. Women who had HIV testing experience/with partner were grouped as "Yes"; otherwise they were regarded as "No". PMTCT knowledge was assessed using five questions, *'*Have you ever heard about mother-to-child HIV transmission?', 'Can an HIV infected mother infect her baby with HIV during pregnancy/delivery/breast feeding?' 'Yes, No and Unsure/I don't know'. Women who said yes were categorized as knowledgeable otherwise not knowledgeable. The fifth question was about the prevention of MTCT; 'What measures do you know for the prevention of mother-to-child HIV transmission?'. A list of prevention interventions was presented with a possibility to choose more than one. Accordingly women who chose more than one interventions were considered knowledgeable; otherwise not knowledgeable. A sum score was constructed from the five items (range 5 to 10). The higher the score the more knowledgeable the woman is.

The third phase actual HIV testing was assessed objectively through information obtained from the PMTCT log books, "What kind of pre-test counselling offered to the woman (individual or group)?", "Did the woman receive HIV testing?", " What was the HIV test result?" and "Did the woman receive post-test counselling?".

### Statistical analyses

The quantitative data was double entered in excel spreadsheet and checked for inconsistencies by creating a check file. Then the data was transferred to SPSS version 17 for analysis. Descriptive statistics, Pearson Chi Square tests, were used to describe and to explore baseline differences in socio-demographic, obstetric and other characteristics between women attending public- and private health facilities. Fisher's Exact Tests were used to check baseline differences in demographic, obstetric and other characteristics between women who completed their follow up and those who did not. Internal consistency reliability was conducted using Chronbach's alpha. Pearson's correlation was used to examine bivariate linear relationship between intention and the TPB variables, PMTCT knowledge, descriptive norms and previous HIV testing experiences. Multiple linear regression analysis was applied to explain intention from TPB and external variables to calculate R^2 ^and β values, separately for women attending private- and public health care facilities and separately for each health centre and hospital. Forward conditional logistic regression analysis was applied to explain actual HIV testing, to examine the relative contribution of the TPB and external variables and to assess the fit of the model in terms of Nagelkerke R^2 ^and to control for potential confounding effect. Despite using a cohort design, Odds Ratio (OR) was used as an effect measure taking into consideration the fixed follow up time, little losses to follow up and the low prevalence of not tasting for HIV in antenatal settings. "In cohort studies on acute disease without induction period and a short time of follow up, like outbreaks, the risk of disease can be estimated directly using the cumulative incidence given a fixed cohort with fixed period of follow up and a low fraction of drop-outs" [37]. Moreover, under rare disease assumption, the OR may be an acceptable approximation of the risk ratio [38-40]. To examine possible moderation effect of type of pre-test counselling upon the intention-behaviour relationship, a two way interaction term between intention and type of pre-test counselling was added to the regression model and tested for statistical significance. Sensitivity, specificity and predictive values were calculated to examine the nature of intention-behaviour relationship. P-values < 0.05 was considered significant and for the odds ratio, 95% confidence interval (CI) was used.

## Results

### Sample profile

Among the 3082 first time antenatal attendees approached, 49 (3.5%) refused to participate in the study, where most of them claimed that they had no time. A total of 3033 women completed questionnaire interviews in the second phase of the data collection. Of these 2928 (96.5%) women completed their follow up to HIV testing in the third phase. Hundred and five women did not complete their follow up, 98 were not given pre-test counselling and 7 did not have complete information regarding pre-test counselling.

Table 1 depicts the characteristics of the participants according to type of health care facility attended. Women attending public facilities were younger than women attending private facilities. The majority of the women (38.7%) attending public health care facilities had education less than 5^th ^grade, whereas 95.7% women attending private health care facilities had education above 9 grade. A total of 73.8% of the women investigated received group pre-test information. The majority (77.1%) of the women attending public health care facilities received group pre-test information, whereas 80.6% of the women attending private health care facilities received individual pre-test counselling.

**Table 1:** Frequency distribution of women's soci-demographic and obstetric characteristics, PMTCT knowledge and HIV testing experience by public- and private health care facilities

*Variable*	*Whole sample* *N = 3033* *n (%)*	*Public facilities* *n = 2751* *n (%)*	*Private facilities* *n = 282* *n (%)*	*P-Value*
**Age in years**				< 0.001
15-24	1444(48.4)	1378(50.9)	66(23.8)	
> 25	1541(51.6)	1330(49.1)	211(76.2)	
**Education/grades completed**				< 0.001
0 - 4	1061(35.1)	1060(38.7)	1(0.4)	
5 - 8	857(28.4)	846(30.9)	11(3.9)	
> 9	1103(36.5)	834(30.4)	269(95.7)	
**Number of pregnancies**				< 0.01
1	1524(50.3)	1357(49.3)	167(59.2)	
> 2	1508(49.7)	1393(50.7)	115(40.8)	
**PMTCT knowledge**				< 0.001
Knowledgeable	2240(76.5)	1987(75.0)	253(91.0)	
Not knowledgeable	687(23.5)	662(25.0)	25(9.0)	
**Previous HIV testing experience**				< 0.001
Yes	2450(81.0)	2185(79.7)	265(94.0)	
No	575(19.0)	558(20.3)	17(6.0)	
**Previous HIV testing experience** **with partner**				< 0.001
Yes	1673(57.2)	1465(55.3)	208(75.6)	
No	1250(42.8)	1183(44.7)	67(24.4)	
**Type of pre-test counselling**				< 0.001
Group pre-test information	2130(73.8)	2098(77.1)	32(19.4)	
Individual counselling	757(26.2)	624(22.9)	133(80.6)	

There was no statistically significant difference between women who completed their follow up and those lost to follow up with respect to age, education, knowledge of PMTCT, previous HIV testing experience and previous HIV testing experience with partner (p > 0.05) (not shown in table).

### The TPB variables

Cronbach's alpha for the TPB constructs ranged from 0.75 for attitude and subjective norm to 0.92 for intention (Table 2). In general, the women attending both private- and public health care facilities had high intention, favourable attitude, perceived strong normative pressure, and perceived less barriers to undertake HIV testing. There were statistically significant differences between women attending public- and private health care facilities across all theoretical constructs considered (p < 0.01), except for descriptive norms. Thus, women attending public health care facilities reported slightly stronger intention, more favourable attitude and stronger perceived normative pressure to undergo HIV testing, compared to their counterparts attending private health care facilities (Table 2).

**Table 2:** Descriptive statistics for TPB variables and external variables by public and private health care facility

*Construct*	*Whole sample, N = 3033*				*Public, n = 2554*		*Private, n = 266*		*P-value*
	
	α	Items (Range)	Mean	SD	Mean	SD	Mean	SD	
Intention	0.92	3(3-15)	11.1	3.53	11.3	3.50	9.2	3.21	0.000
Attitude	0.75	3(3-15)	13.3	1.43	13.4	1.43	12.3	0.99	0.000
Subjective norm	0.75	4(4-20)	15.1	2.94	15.2	2.98	13.9	2.21	0.000
Descriptive norm		1(1-5)	3.3	1.10	3.3	1.12	3.3	0.88	0.538
Perceived barriers	0.71	4(4-20)	9.2	2.88	9.3	2.97	8.7	1.66	0.002

### Explaining intention to test for HIV

The bivariate relationships between intention and the TPB variables, descriptive norm, PMTCT knowledge, previous HIV testing were examined using Pearson's product-moment correlation coefficient, separately for public- and private health care facility attendees (Table 3). Intention was significantly and positively associated with attitude, subjective norm, descriptive norm, PMTCT knowledge, previous HIV testing experience and previous HIV testing experience with partner among public health care facility attendees (the correlation above the diagonal in Table 3). Similarly, intention was significantly and positively associated with attitude, subjective norm and descriptive norm among women attending private health care facility (the correlation below the diagonal in Table 3). By contrast, intention was negatively associated with perceived barrier both in public and private facility attendees, yet the association was not significant among private facility attendees.

**Table 3:** Pearson's correlation between TPB variables and external variables in public and private health care facilities

*Variable*	*1*	*2*	*3*	*4*	*5*	*6*	*7*	*8*
1. Intention	1	0.20(**)	0.27(**)	0.19(**)	-0.11(**)	0.13(**)	0.11(**)	0.10(**)
2. Attitude	0.24(**)	1	0.19(**)	0.05(**)	-0.15(**)	0.16(**)	0.12(**)	0.07(**)
3. Subjective norm	0.41(**)	0.24(**)	1	0.45(**)	-0.14(**)	0.05(*)	0.09(**)	0.10(**)
4. Descriptive norm	0.26(**)	0.11	0.60(**)	1	-0.18(**)	0.05(**)	0.08(**)	0.09(**)
5. Perceived barrier	-0.01	-0.03	0.12(*)	0.02	1	0.03	-0.12(**)	-0.10(**)
6. PMTCT Knowledge	0.17(**)	0.04	0.20(**)	0.13(*)	-0.03	1	0.13(**)	0.08(**)
7. Previous HIV testing experience	0.08	0.04	-0.00	0.05	-0.23(**)	0.18(**)	1	0.56(**)
8. Previous HIV testing experience with partner	0.04	0.04	-0.01	-0.05	-0.17(**)	0.18(**)	0.52(**)	1

**p < 0.001, *p < 0.05 (2 tailed)

*NB- The correlation above the diagonal is for public health care facility attendees and the one below the diagonal is for private health care facility attendees*.

All variables that were statistically significantly associated in the bivariate analysis (Table 3) were entered into the multiple linear hierarchical regression models. Among women attending public health care facilities, previous HIV testing experience, previous HIV testing experience with partner and PMTCT knowledge entered in the first step accounted for 2.3% of the variance in intention to test for HIV [ΔR^2 ^= 0.023, F change = 18.90 (3, 2443), p < 0.001]. Previous HIV testing experience (β = 0.056, p < 0.05) and PMTCT knowledge (β = 0.125, p < 0.001) were significantly associated with intention. Entering the TPB variables and descriptive norms in the second step, increased the explained variance with 9.2% [(ΔR^2 ^= 0.092, F change = 63.36 (4, 2439), p < 0,001)]. In the final second step, subjective norm (β = 0.20, p < 0.001) had the strongest impact followed in descending order by attitude (β = 0.14, p < 0.001), PMTCT knowledge (β = 0.093, p < 0.001) and descriptive norm (β = 0.08, p < 0.001). Among women attending private health care facilities, PMTCT knowledge (β = 0.08, p > 0.05) entered in the first step accounted for 2.5% variance in intention [(ΔR^2 ^= 0.025, F change = 6.74 (1, 261), p < 0.01)]. Entering the TPB components and descriptive norms in the second step increased the explained variance with 16.4% [(ΔR^2 ^= 0.164, F change = 13.0 (4, 257), p < 0,001)]. In the final step, the impact of PMTCT knowledge did not remain statistically significant. Subjective norm (β = 0.32, p < 0.001) had the strongest impact followed by attitude (β = 0.125, p < 0.05) (Table 4).

**Table 4:** Intention to test for HIV regressed on previous HIV testing, PMTCT knowledge, TPB variables and descriptive norm

		*Public facility*			*Private facility*		
		
*Step*	Variable	β final Step (Sig)	**R**^**2**^	**R**^**2**^ Change	β final Step (Sig)	**R**^**2**^	**R**^**2**^ Change
**Step I**	Previous HIV testing experience	0.03(0.14)					
	Previous HIV testing experience with partner	0.14(0.53)					
	PMTCT Knowledge	0.09(0.00)			0.08(0.15)		
			0.023	0.023		0.025	0.025
**Step II**	Attitudes	0.14(0.00)			0.12(0.03)		
	Subjective norms	0.20(0.00)			0.32(0.00)		
	Descriptive norm	0.08(0.00)			0.07(0.34)		
	Perceived barriers	-0.04(0.07)					
			0.115	0.092		0.19	0.164

Stratified analyses by PMTCT centre showed that, the explained variance in intended HIV testing from the TPB variables and descriptive norm ranged from 7.7% to 32.5%, whereby attitude, subjective norm and descriptive norm explained most of the variance in intention (not shown in table).

### Explaining HIV testing behaviour

Of the 2928 women who completed their follow up in phase III, 128 (4.4) did not test for HIV. Table 5 depicts the adjusted odds ratios and 95% CI for actual HIV testing by intention, perceived barriers and the external variables. Socio-demographic and obstetric variables, PMTCT knowledge, previous HIV testing experience and type of pre-test counselling were entered in a first step. In the first step, the chi-square was significant (χ^2 ^= 40.36 [8], p < 0.001) and explained 7% of the variance in actual HIV testing according to Nagelkerke R^2 ^(0.07, p < 0.001). Intention and perceived barrier were entered in the second step and increased the explained variance with 2.4%. The full model containing all variables was statistically significant and explained a total of 9.4% of the variance in HIV testing (Nagelkerke R^2 ^= 0.094, p < 0.01). In the final model, women who had over 8 grade of education were less likely to test for HIV compared to women with lower level of education. Similarly women who received individual pre-test counselling were 1.85 times [95% CI (1.14, 3.03)] less likely to test for HIV compared to women who received group pre-test information. Moreover, women who had low intention to test for HIV were 2.4 times [95% CI (1.45, 3.85)] less likely to test for HIV compared to their high intending counterparts. To check whether type of pre-test counselling moderated the relationship between intention and actual HIV testing, an interaction term (intention*type of pre-test counselling) was entered in a third step, after controlling for all other variables in the model. This interaction term did not add significantly to the model (Nagelkerkes R^2 ^= 0.098, P > 0.05) (Table 5).

**Table 5:** Actual HIV testing regressed upon TPB and other variables. OR and 95% CI

*Variable*	*Tested for HIV*			
			
	No n(%)	Yes n(%)	*Step 1* *Nagelkerke* *R^2 ^= 0.070* *OR (95% CI)* *n = 2586*	*Step 2* *Nagelkerke* *R^2 ^= 0.094* *OR (95% CI)* *n = 2586*
**Age**				
15-24	53(3.7)	1366(96.3)	1	
> 25	72(4.9)	1389(95.1)	0.99 (0.58, 1.70)	
**Education**				
0 - 4	21(2.0)	1032(98.0)	1	1
5 - 8	17(2.0)	833(98.0)	1.08(0.47, 2.46)	1.06(0.46, 2.43)
> 9	87(8.6)	926(91.4)	0.28(0.15, 0.53)	0.29(0.15, 0.56)
**PMTCT knowledge**				
Knowledgeable	96(4.5)	2053(95.5)	1	
Not knowledgeable	30(4.5)	643(95.5)	0.66(0.38, 1.16)	
**Number of pregnancies**				
1	71(4.9)	1385(95.1)	1	
> 2	57(3.9)	1414(96.1)	0.94(0.56, 1.58)	
**Previous HIV testing experience**				
Yes	106(4.5)	2249(95.5)	1	
No	22(3.9)	543(96.1)	0.58(0.28, 1.20)	
**Previous HIV testing experience** **with partner**				
Yes	83(5.2)	1522(94.8)	1	
No	38(3.1)	1176(96.9)	1.38(0.75, 2.56)	
**Type of pre-test counselling**				
Individual counselling	36(4.8)	720(95.2)	1	1
Group pre-test information	46(2.2)	2078(97.8)	1.92(1.18,3.12)	1.85(1.14,3.03)
**Intention**				
Low	59(6.0)	925(94.0)		1
High	69(3.5)	1875(96.5)		2.38(1.45,3.85)
**Perceived barrier**				
Yes	27(3.6)	717(96.4)		1
No	101(4.7)	2052(95.3)		1.41(0.83,2.38)

Based on cross tabulation of intention and behaviour, epidemiological indices were calculated to examine the nature of the intention -behaviour link. As shown in Table 6, sensitivity was 67% indicating that the majority of the women who tested for HIV had high intention to do so. The specificity of 46% indicated that even women who were not tested had high intention to test. The positive predictive value was 96% indicating that, high intention was a strong predictor of being tested for HIV. The negative predictive value was 6% indicating that, among the women who had low intention the majority of them were tested (Table 6).

**Table 6:** Levels of intentions and actual HIV testing with associated values of epidemiological indices

*Intention *	*HIV testing*					*Predictive value*	
	Yes	No	Total	*Sensitivity*	*Specificity*	Positive	Negative
High	1875	69	1944	67%	46%	96%	6%
Low	925	59	984				
Total	2800	128	2928				

## Discussion

This study applied an extended version of the TPB framework to explain intended- and actual HIV testing among pregnant women upon their first time antenatal care attendance. The cognitive variables and previous HIV testing experience explained 11.5% and 19% of the variance in intentions to test for HIV among women attending public- and private health care facilities, respectively. As indicated by the significant effect of PMTCT knowledge upon behavioural intention, the TPB appeared less sufficient to account for intended HIV testing in public- than in private health care facility attendees. Nevertheless, most external variables did not maintain their statistical significance when the TPB components were considered, suggesting that the TPB provided a fairly accurate description of the intention formation process related to HIV testing in both groups considered. Compared to recent meta analytical reviews, suggesting a predictive power of 39% in behavioural intentions, the present results indicate a fit below the optimal level [21]. The explained variance in intention from the TPB variables was also lower than what has been reported in previous studies from non-occidental settings focusing on voluntary HIV counselling and testing [25], condom use [26] and motivation to learn about HIV/AIDS [24]. Yet, the present figures compare more favourably to TPB studies in other behavioural domains [22,41].

The TPB suggests that changing intentions can be accomplished by influencing attitudes, subjective norms and perceived behavioural control. Thus, to design effective interventions, the relative importance of attitudes, subjective norms and perceived behavioural control in explaining intention becomes vital [1]. As judged from the standardized regression coefficients, this study indicates that the decision to test for HIV upon first time antenatal care attendance was primarily under the control of subjective norms and attitudes, whereas descriptive norms and perceived barriers were less important. This means that pregnant women decided to test for HIV if they perceived a positive normative pressure and if they anticipated more positive than negative personal consequences following testing. In agreement with our finding, in a review that evaluated barriers to HIV testing, perceived benefit of taking an HIV test was an important HIV test promoting factor [42]. This appears to imply that educational messages to increase women's motivation should target attitudinal beliefs women hold about the consequences of HIV testing. Such messages could positively influence their attitudes towards HIV testing, either by changing the strength of beliefs or by introducing new beliefs. In addition messages based on normative pressure might be an effective way to convince pregnant women to test for HIV. In previous studies among Tanzanian school teachers and homeless people in the United States of America, intentions to seek voluntary HIV testing are strongly influenced by attitude and subjective norm [25,43]. In the Ethiopian context, attitude and subjective norms have also been identified to be the strongest predictors of intended condom use among youths [26] and strong predictors of university students' motivation to learn about HIV/AIDS [24]. Moreover, in a meta-analysis of screening attendance generally, subjective norm appeared to be a strong predictor of intentions in the prenatal context. This suggests that decisions made in this context does not only concern the pregnant women, but also husband, the unborn child and significant others [44]. Thus, interventions to improve uptake of PMTCT services should target the family as a whole rather than focusing only on the individual pregnant women. In this regard partner involvement in HIV testing might be an important strategy to be strengthened particularly to manage fear of disclosure, one of the salient barriers identified in the FGDs.

Actual HIV testing was significantly associated with intention; however this was not the case with perceived barriers (Table 5). The significant intention-behaviour link is in accordance with the proposition from the TPB [1]. Our finding is also consistent with findings from a review and meta-analysis on screening attendance that reported intentions to be the strongest predictor of participation in screening [1,44,45]. In general, the power of the TPB in explaining actual HIV testing was weak, accounting for only 2.4% of the total variance in HIV testing. Various factors could explain this weak association. First, it could be due to the low variability in the data, where 95.2% of the women being tested. Second, there could be some shared variances being lost due to measurement discordance [21], as the TPB variables were self reported whilst the HIV testing was assessed objectively. Third, it could be attributed to a more unstable measure of behavioural intention. Although the internal consistency reliability of intention was satisfactory, this is a measure of on the spot reliability. What is important is that intention might change across time since events crop up as the time between the assessment of intention and behaviour increases. In this study the time interval between measuring intention and actual HIV testing was short but pre-test counselling/information offered to participants probably contributed to a change in women's initial intention not to test. Finally, the cognitive determinants seem unable to take account of the full range of variables that could affect the behavioural outcome, including routines of the facilities and the asymmetric power relationship between PMTCT providers and the antenatal attendees.

As demonstrated by the epidemiological indices in Table 6, a possible reason for the intention behaviour gap was a large proportion of non-intenders who actually tested or the very low proportion of true negatives (only 6%). In a previous study of physical activity, false positives (inclined abstainers) were identified to be the most important cause of the gap between intended exercise and actual exercise behaviour [46]. It was also shown that women who attended individual pre-test counselling were 1.85 times less likely to test for HIV compared to women who attended group pre-test information (Table 5). Gruskin, et al., (2008) pointed out that insufficient pre-test information given to women in a routine opt-out approach is detrimental to ensuring informed consent as well as to coping with a positive HIV test. More studies from Sub-Saharan Africa revealed that the information given to pregnant women in the group pre-test sessions was inadequate and, mainly focused on getting the women tested without enabling them to opt out if they did not intend to test [10,11,13]. From a practical point of view, the present analysis suggests that in order to promote HIV testing at first time antenatal visiting, it will be important to motivate non-intenders but also to focus on women who have positive intentions but still do not test for HIV. In addition it is important to ensure that the fundamental principle of informed consent is not violated and that testing follows an informed choice.

To our knowledge this is the first study that applied the TPB to explain intention and actual HIV testing in antenatal care settings. One of the strengths of the study is the use of mixed methods where the qualitative FGDs had informed the quantitative questionnaire. Since all the interviews were conducted at health care facilities using a face-to-face interview, the possibility of social desirability bias cannot be excluded. There could be a possibility of inter-rater variability as we have used 17 field assistants. To minimize the inter-rater variability, field assistants having similar educational level were recruited and trained as a group for two days. In addition, all field assistants participated in pretesting of the questionnaire under close supervision of the principal investigator. A further strength of the study was that, the actual HIV testing behaviour of the participants was obtained objectively from PMTCT log books.

## Conclusions

This study has brought a new prospect to optimize the effectiveness of the routine opt-out HIV testing policy by focusing upon cognitive determinants of intended and actual HIV testing in antenatal settings. According to the findings, pregnant women's intention to test for HIV upon first time antenatal care attendance was based on their normative expectations and the likely consequences following testing, in that order. Thus, women intended to test for HIV if they perceived social support, but also if they anticipated positive consequences accruing from testing. The TPB variables were less sufficient in predicting actual HIV testing, mainly due to a high proportion of non- intenders who completed HIV testing. This suggests that in the routine opt-out testing, women's lack of intention to test may not matter for the end result. Tailored behaviour change communication might be a way forward to facilitate informed and voluntary HIV testing decisions. Attempts aimed at increasing women's motivation to test for HIV should strengthen their intention to do so through informed awareness accompanied with social approval and strengthened conviction that HIV testing will provide mostly positive consequences for them. Women's rights to opt-out from testing should be highlighted and sufficient pre-test information provided to strength PMTCT programs.

## List of abbreviations

ARV: Antiretroviral; CI: Confidence Interval; CSA: Central Statistical Authority; GAMET: Global HIV/AIDS Monitoring and Evaluation Team; FGD: Focus Group Discussion; FHAPCO: Federal HIV/AIDS Prevention and Control Office; MOH: Ministry of Health; MTCT: Mother-to-Child HIV Transmission; OR: Odds Ratio; PBC: Perceived Behavioural Control; PMTCT: Prevention of Mother-to-Child HIV Transmission; TPB: Theory of Planned Behaviour; WHO: World Health Organization; UNAIDS: Joint United Nations Programmes on HIV/AIDS; UNDP: United Nations Development Programme.

## Competing interests

The authors declare that they have no competing interests.

## Authors' contributions

AHM prepared the study proposal, collected and analyzed the data, interpreted the findings and wrote the manuscript. ANÅ was involved in developing the questionnaire, analyzing the data and revising the manuscript. MMS was involved in training the data collectors, preliminary data analysis and revising the manuscript. KMM was involved in developing the study proposal and revising the manuscript. All authors have read and approved the final manuscript.

## Author information

Corresponding author information - AM is from Ethiopia, currently doing her PhD at the Centre for International Health, University of Bergen, Norway.

## Pre-publication history

The pre-publication history for this paper can be accessed here:

http://www.biomedcentral.com/1472-6963/11/196/prepub
